# VCP/p97 inhibitor CB-5083 modulates muscle pathology in a mouse model of VCP inclusion body myopathy

**DOI:** 10.1186/s12967-021-03186-6

**Published:** 2022-01-08

**Authors:** Cheng Cheng, Lan Weiss, Henri Leinonen, Alyaa Shmara, Hong Z. Yin, Timothy Ton, Annie Do, Jonathan Lee, Lac Ta, Eshanee Mohanty, Jesse Vargas, John Weiss, Krzysztof Palczewski, Virginia Kimonis

**Affiliations:** 1 Division of Genetics and Genomic Medicine, Dept. of Pediatrics, UC Irvine, Irvine, CA USA; 2 Gavin Herbert Eye Institute, and the Department of Ophthalmology, Center for Translational Vision Research, University of California, Irvine, Irvine, CA USA; 3 Department of Neurology, University of California, Irvine, Irvine, CA USA; 4 Cleave Therapeutics, Inc., San Francisco, CA USA; 5 Department of Physiology & Biophysics, University of California, Irvine, Irvine, CA USA; 6 Department of Chemistry, Molecular Biology & Biochemistry, University of California, Irvine, Irvine, CA USA; 7 Department of Pathology, University of California, Irvine, Irvine, CA USA; 8 Department of Environmental Medicine, University of California, Irvine, Irvine, CA USA

## Abstract

**Background:**

Pathogenic gain of function variants in Valosin-containing protein (*VCP*) cause a unique disease characterized by inclusion body myopathy with early-onset Paget disease of bone and frontotemporal dementia (also known as Multisystem proteinopathy (MSP)). Previous studies in drosophila models of VCP disease indicate treatment with VCP inhibitors mitigates disease pathology. Earlier-generation VCP inhibitors display off-target effects and relatively low therapeutic potency. New generation of VCP inhibitors needs to be evaluated in a mouse model of VCP disease. In this study, we tested the safety and efficacy of a novel and potent VCP inhibitor, CB-5083 using VCP patient-derived myoblast cells and an animal model of VCP disease.

**Methods:**

First, we analyzed the effect of CB-5083 in patient-derived myoblasts on the typical disease autophagy and TDP-43 profile by Western blot. Next, we determined the maximum tolerated dosage of CB-5083 in mice and treated the 2-month-old VCP^R155H/R155H^ mice for 5 months with 15 mg/kg CB-5083. We analyzed motor function monthly by Rotarod; and we assessed the end-point blood toxicology, and the muscle and brain pathology, including autophagy and TDP-43 profile, using Western blot and immunohistochemistry. We also treated 12-month-old VCP^R155H/+^ mice for 6 months and performed similar analysis. Finally, we assessed the potential side effects of CB-5083 on retinal function, using electroretinography in chronically treated VCP^R155H/155H^ mice.

**Results:**

In vitro analyses using patient-derived myoblasts confirmed that CB-5083 can modulate expression of the proteins in the autophagy pathways. We found that chronic CB-5083 treatment is well tolerated in the homozygous mice harboring patient-specific VCP variant, R155H, and can ameliorate the muscle pathology characteristic of the disease. VCP-associated pathology biomarkers, such as elevated TDP-43 and p62 levels, were significantly reduced. Finally, to address the potential adverse effect of CB-5083 on visual function observed in a previous oncology clinical trial, we analyzed retinal function in mice treated with moderate doses of CB-5083 for 5 months and documented the absence of permanent ocular toxicity.

**Conclusions:**

Altogether, these findings suggest that long-term use of CB-5083 by moderate doses is safe and can improve VCP disease-associated muscle pathology. Our results provide translationally relevant evidence that VCP inhibitors could be beneficial in the treatment of VCP disease.

**Supplementary Information:**

The online version contains supplementary material available at 10.1186/s12967-021-03186-6.

## Introduction

Multisystem proteinopathy caused by variants in the *VCP* gene is characterized by inclusion body myopathy, Paget disease of the bone, and frontotemporal dementia. The disease predominantly affects the muscle, bones, and the central nervous system (CNS). VCP disease is a rare neuromuscular disease that has been diagnosed in several hundred people worldwide [1–3]. Among these patients, myopathy occurs in 80–90% of individuals with a mean onset of 42 years of age [2, 4]. The muscle weakness typically starts in the proximal girdle muscle involving the pelvis and shoulder and progresses distally. In addition, Paget disease of bone occurs in 49% of patients with a similar age of onset as myopathy and affects the vertebral column, pelvis, scapulae, and skull [5]. As a result, patients often suffer from pain, bone deformities, and fractures. Furthermore, premature frontotemporal dementia (FTD) is observed in 27% of patients with a mean onset of 57 years of age; and amyotrophic lateral sclerosis is seen in 9% of patients [3]. Affected individuals manifest clinical signs of neurodegeneration in the frontal and temporal lobes, characterized by comprehension deficits, dysnomia, and social unawareness. VCP disease is a devastatingly progressive disease, and patients typically die in their 40 s to 50 s from cardiac or respiratory failure [6]. Despite a well-characterized genetic cause, there are currently no effective treatments available, except for symptomatic treatment and bisphosphonates including zoledronic acid, pamidronate, risedronate and alendronic acid which are first line treatments, and can help symptoms of bone pain as a result of inhibition of overactive osteoclasts for the Paget component of the disease [7].

VCP is an AAA ATPase associated with a broad range of cellular activities, including the ubiquitin–proteasome system (UPS), endoplasmic reticulum-associated degradation of proteins (ERAD), DNA repair, autophagosome maturation, and mitochondrial fusion [6, 8–13]. In vitro analysis of VCP mutants has shown pathologically enhanced ATPase enzymatic activity [14–16]. Additionally, excision of the mutant allele in the heterozygous mouse model of VCP disease, which contains the most common patient-specific mutation R155H, has been shown to improve the disease pathology [17]. Recent studies in drosophila models carrying gain of function pathogenic VCP mutations have shown severe mitochondrial fusion defects, and increased degradation of mitofusins (MFN1 and MFN2) [18]. Researchers were able to reduce these mitochondrial defects using early generations of VCP inhibitors, NMS873 and ML240 [18]. Even though these compounds have poor pharmaceutical properties for clinical use, the previous studies provided proof-of-concept that normalizing VCP hyperactivity using pharmacological inhibitors could be an effective treatment. However, translationally more relevant evidence from mammalian disease models has been lacking.

CB-5083 is a novel VCP inhibitor that preferentially targets the D2 ATPase domain [19–22]. It inhibits VCP reversibly and competitively. CB-5083 displays high specificity and potency with an IC_50_ of 11 nM for VCP. This renders CB-5083 an excellent candidate for testing the hypothesis that normalization of upregulated VCP activity is therapeutic in a translationally relevant mouse model [23]. A previous study on rodent cancer xenograft models with elevated VCP expression showed that CB-5083 can significantly decrease tumor progression and growth [23]. Based on these preclinical results Cleave Therapeutics (previously Cleave Biosciences) Inc. conducted a Phase 1 trial of CB-5083 involving 84 subjects with solid tumors and multiple myeloma. This clinical trial was terminated due to adverse off-target effects on visual function. Even though the trial was not successful in achieving the desired endpoint, it did demonstrate CB-5083 as generally safe and otherwise well tolerated at moderate doses that are subtherapeutic for application in oncology but could potentially be beneficial in the context of VCP disease.

Here, we provide the first proof-of-concept study using the VCP-inhibitor CB-5083 for the treatment of VCP disease. The effect of CB-5083 was tested with patient-derived myoblasts and with the VCP R155H knock-in mice that recapitulate clinical manifestations of VCP disease [24, 25]. Chronic administration of CB-5083 was well tolerated in mice as determined by longitudinal weight monitoring and end-point serum biochemical analyses. CB-5083 displayed significant modulatory effects on the expression levels of disease biomarkers including TDP-43, autophagy markers p62, and the lysosomal damage marker, transcription factor EB (TFEB). The muscle morphology in the mice treated with CB-5083 was also improved. Importantly, we showed that chronic CB-5083 treatment had a transient effect on retinal dysfunction. Altogether, our results indicate that CB-5083 at reduced doses than utilized for treatment of malignancies is safe for long-term use and may benefit patients with VCP myopathy.

## Results

### CB-5083 modulated autophagy pathways in patient-derived primary myoblasts

**Fig. 1:**
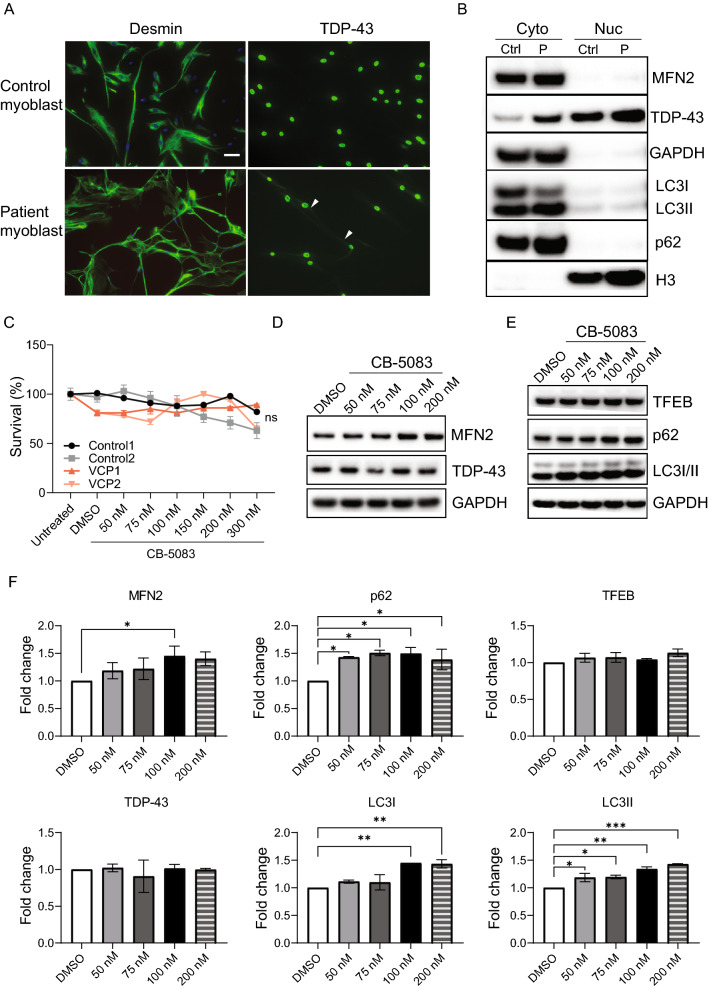
Evaluation of CB-5083 with patient-derived primary myoblasts. **A** Immunohistochemical analysis indicates that primary myoblasts express the myoblast marker desmin (green). TDP-43 cytoplasmic signaling (green) is present in patient myoblasts (scale = 20 µm, nuclear stain = blue). Arrowhead points to the cytoplasmic TDP-43 signals. **B** Analysis of nuclear-cytoplasmic fractions reveals that cytoplasmic p62 levels are higher in the patients’ myoblasts (P) compared to control myoblasts (Ctrl). GAPDH and H3 were used as loading controls for cytoplasmic and nuclear fractions, respectively. Western Blot experiments were repeated in two patients and two control myoblasts for two times, and the results were consistent. **C** Patient and control myoblasts tolerated serial treatment with CB-5083 at concentrations up to 300 nM. The MTT analysis was performed in two control and two patient myoblasts for three time. No significant differences were observed across the four samples (*P* = 0.557, one-way ANOVA, followed by Fisher’s LSD). **D**, **E** Dose–response effects of CB-5083 on biomarkers in patient myoblasts. Autophagy markers, LC3I/II and p62, and MFN2 were upregulated upon CB-5083 treatment. TDP-43 and TFEB did not change. **F** Quantification of Western blots from two independent patient myoblast lines was shown in (**D**, **E**). Western blot was repeated at least twice. Statistical analysis was performed by one-way ANOVA followed by Fisher’s LSD test. *P < 0.05. **P < 0.01. ***P < 0.001

We first analyzed CB-5083’s dose–response in cellular models of VCP disease, namely patient-derived myoblast cells carrying the VCP p.R93C and p.R155H variants. These studies also served to investigate the acute effects of CB-5083 treatment. We first validated the control and patient cells as myoblasts with their strong desmin positive signals and confirmed the pathology of the patient myoblasts (Fig. 1A). TDP-43 was highly expressed in the nucleus of both cell lines, however, cytoplasmic TDP-43 was found only in the VCP disease patient-derived myoblasts in 2% to 15% of the cells (Fig. 1A, Additional file 1: Fig. S1C). By using cytoplasmic-nuclear fractionation and immunoblotting, we confirmed that TDP-43 was slightly elevated in the cytoplasmic compartment of VCP-mutant patient derived myoblasts (Fig. 1B). Because of the variability between the two patient-derived myoblasts, the cytoplasmic signals observed in patient-derived myoblasts were not significantly different from the control myoblasts statistically (Additional file 1: Fig. S1A, C), albeit the cytoplasmic TDP-43 levels were increased and were only detected in the patient myoblasts (Additional file 1: Fig. S1C). It is plausible that the variability of TDP-43 cytoplasmic signals can be due to potential disease state and the age of the patients. Autophagy markers p62 were also elevated in the cytoplasmic fraction of patient myoblasts (Fig. 1B), as observed in patient muscle. Interestingly, there was a trend toward increased LC 3II/I ratio (Additional file 1: Fig. S1B). We speculate that the patient-derived myoblasts could display arrested autophagy. Altogether, these results confirmed that our primary patient-derived myoblasts harbor the TDP-43 phenotype and are a useful model of the disease. No differences were noted in the expression level of mitofusin between patient and control myoblasts (Fig. 1B).

We next examined the effects of CB-5083 on these patient myoblasts at doses ranging from 50 to 300 nM for 5 consecutive days. Both control and patient-derived myoblasts tolerated CB-5083 well up to 300 nM dosage with equally high survival rates demonstrated by MTT assay (Fig. 1C). Upon VCP inhibition by CB-5083, we observed a significant increase in levels of MFN2 in the myoblasts (Fig. 1D, F). This is in agreement with published data showing that VCP facilitates the degradation of mitofusin [18]. Interestingly, when we treated the patient-derived myoblasts with 75 nM of CB-5083 for 5 days, followed by collecting the nuclear and the cytoplasmic fraction to test for TDP-43, we did not observe differences upon treatment (Additional file 1: Fig. S1D, E). We speculate a 5-day treatment of CB-5083 might be too short to reveal any pathological amelioration in TDP-43 levels. VCP inhibition by CB-5083 and other competitive inhibitors has been shown to modulate autophagy by either activating autophagy [26, 27] or reducing autophagasome formation [28]. Here, we observed an increase in the expression of autophagic markers p62 and LC3I/II, while levels of VCP, and the lysosomal damage marker TFEB were not altered (Fig. 1E, F). These data suggested a modulatory effect of CB-5083 at these doses in multiple cellular pathways. In view of the observed benefits in a drosophila model of VCP disease [18] and the safety of CB5083 on the myoblasts, we proceeded to treatment of the murine model.

### Chronic CB-5083 treatment was well tolerated in VCP R155H knock-in mice

**Fig. 2:**
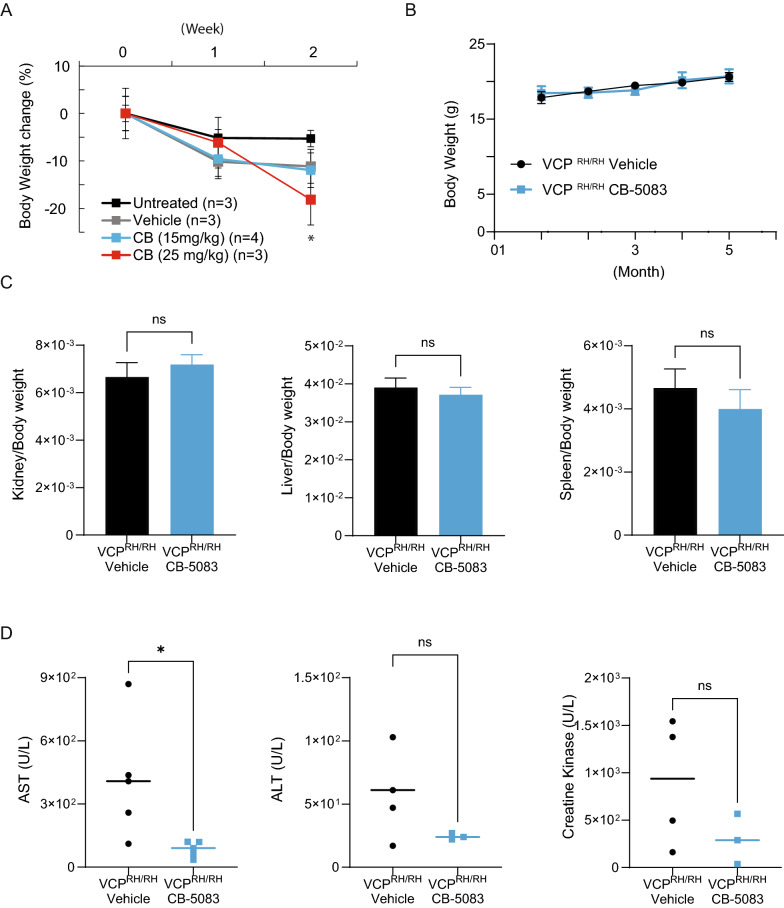
Chronic CB-5083 treatment was well tolerated by VCP^R155H/R155H^ mice. **A** Weight analysis indicated that the maximum tolerated daily dose of CB-5083 is 25 mg/kg in VCP^R155H/+^ mice (i.e., < 20% weight loss). Only CB (25 mg/kg) at 2 weeks was statistically significant (one-way ANOVA, Fisher’s LSD, *P < *0.05*)*.* **B** A steady increase in body weight was observed in VCP^R155H/R155H^ mice during CB-5083 treatment (15 mg/kg) for 5 months. **C** Organ to body weight ratio was unchanged in VCP^R155H/R155H^ mice (vehicle group: n = 7, CB-5083 group: n = 8). **D** Blood toxicology analysis in VCP^R155H/R155H^ mice showed that CB-5083 did not increase liver enzyme levels AST and ALS or creatine kinase levels. AST level was significantly reduced in the CB-5083 treatment group. Statistical analysis was performed by Mann–Whitney U-test: *P < 0.05

Next, we utilized the knock-in mouse model of VCP disease harboring the most commonly found disease-associated mutation, VCP R155H, for our in vivo studies [24, 29]. The heterozygous mice develop progressive muscle weakness at around 12 months, accompanied by cytoplasmic TDP-43 translocation and elevated ubiquitin levels at 15 months of age [30]. We first determined the daily maximum tolerated dosage (MTD) of CB-5083 by administering the drug to VCP^R155H/+^ mice, through daily oral gavage. We found a reduction in body weight when mice were treated with a dose of 25 mg/kg of CB-5083 for 2 weeks (Fig. 2A). However, 15 mg/kg of CB-5083 did not alter mouse body weight compared to the vehicle-treated group. We, therefore, defined 15 mg/kg of daily CB-5083 as a safe and tolerable regimen in these mice.

We next utilized the homozygous VCP^R155H/R155H^ mice for rapid in vivo testing of CB-5083 efficacy. The homozygous VCP^R155H/R155H^ mice typically die before weaning age when maintained on a normal chow diet [31]. However, a continuous lipid-enriched high-fat diet starting from the prenatal period and continued after birth partially reverses lethality in these mice. Roughly 50% of VCP^R155H/R155H^ mice fed on the high-fat diet have a normal life span [31]. Crucially with respect to our current study, these mice also display the typical muscle pathology starting at 3-weeks of age, and muscle weakness starting at 2 months [31], thus making these mice an accelerated disease model of VCP human myopathy [31].

To study safety and efficacy of CB-5083, we first treated the VCP^R155H/R155H^ mice for 5 months starting at 2-months of age. A steady increase in body weight and normal end-point organ weight were observed in both vehicle and CB-5083-treated groups (Fig. 2B, C), indicating that daily CB-5083 (15 mg/kg) administration was also well-tolerated in the young VCP^R155H/R155H^ mice. Additionally, the results obtained from safety labs analyzing end-point serum samples from CB-5083-treated and vehicle-treated VCP^R155H/R155H^ mice, did not reveal an increase in liver enzymes, such as aspartate aminotransferase (AST) and alanine aminotransferase (ALT). In fact, AST level was significantly reduced with CB-5083 treatment and a trend of reduced levels of creatinine kinase (CK), a muscle damage marker, was also observed in mice treated with CB-5083 (Fig. 2D).

### Myofiber pathology was alleviated in VCP^R155H/R155H^ mice with CB-5083 treatment

**Fig. 3:**
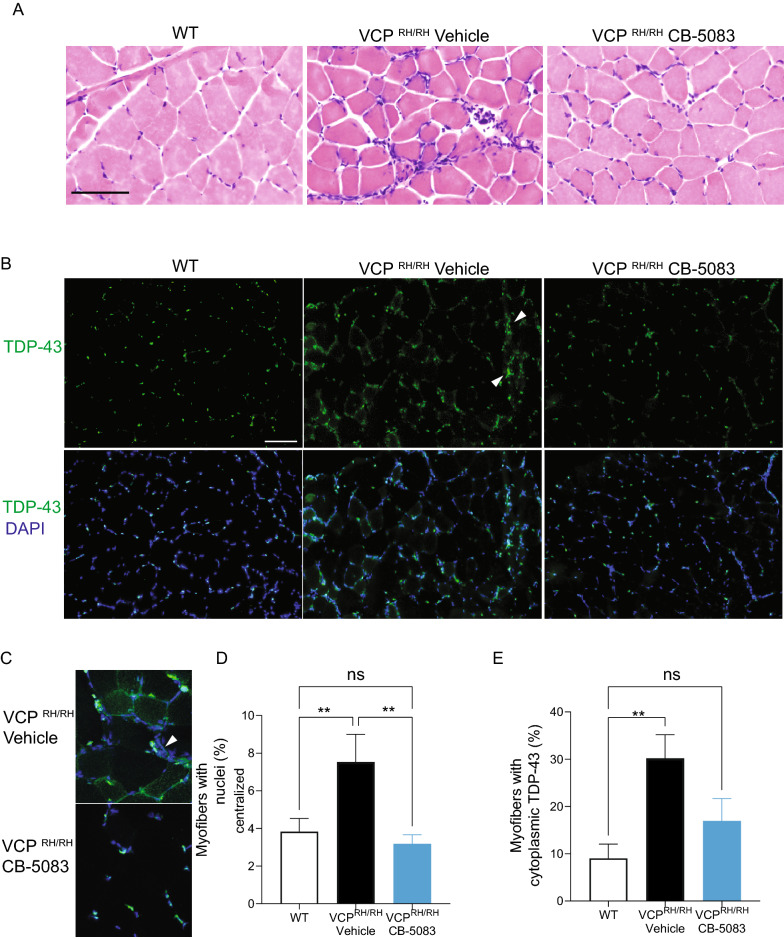
Chronic CB-5083 treatment decreased muscle pathology in VCP^R155H/R155H^ mice. **A** Hematoxylin and eosin (H&E) staining of myofibril structure in WT, and in vehicle and CB-5083-treated VCP^R155H/R155H^ mice after a 5-month treatment. **B**, **C** Immunohistochemical analysis of TDP-43 in muscle sections of WT (n = 4), VCP^R155H/R155H^ mice treated with vehicle (n = 3), and VCP^R155H/R155H^ mice treated with CB-5083 (n = 4). TDP-43 cytoplasmic signals were reduced with less interstitial infiltration upon CB-5083 treatment. Arrowhead points to the interstitially infiltrating cells. **D** Quantification of the centralized nucleus in myofibril sections (n = 3 mice per group). **E** Quantification of myofibers with cytoplasmic TDP-43 signal in WT and VCP^R155H/R155H^ mice upon CB-5083 treatment. Scale = 100 µm. Statistical analysis was performed by one-way ANOVA followed by Fisher’s LSD test: *P < 0.05, **P < 0.01

To assess the effect of CB-5083 on myofiber morphology in VCP^R155H/R155H^ mice, we examined the gross structures of myofibers from mice quadriceps with H&E staining. In the WT mice, myofibers have a homogenous size, and the nuclei are mostly located in the periphery. However, in the VCP^R155H/R155H^ mice, we observed infiltrations of cells into the interstitium of the myofibers (Fig. 3A), and a significant increase in the number of centralized nuclei (Fig. 3D), representing newly regenerated myofibers upon damage. With CB-5083 treatment, the number of centralized nuclei in VCP^R155H/R155H^ mice was significantly reduced compared to vehicle-treated mice (centralized nuclei, WT: 3.9 ± 0.71%, VCP^R155H/R155H^ vehicle: 7.5 ± 1.47%, VCP^R155H/R155H^ CB-5083: 3.2 ± 0.50%, over 300 myofibers from 3 mice per group. ANOVA *P* = 0.01) (Fig. 3A, D).

Since elevated levels of TDP-43, and its translocation from the nucleus to the cytoplasm, have been observed in muscles of the patients and the knock-in VCP mice [32–34], we next analyzed the localization of TDP-43 in the myofibers. We observed cytoplasmic expression of TDP-43 in VCP^R155H/R155H^ mice (Fig. 3B, C). The percentage of myofibers with TDP-43 cytoplasmic signals was significantly reduced with CB-5083 treatment (WT (n = 4): 9.03 ± 3.0%, VCP^R155H/R155H^ vehicle (n = 3): 30.2 ± 5%, VCP^R155H/R155H^ CB-5083 (n = 4): 13.4 ± 5%, Anova *P* = 0.02) (Fig. 3B, E). These data, therefore, suggest that the prolonged 5-month-treatment with CB-5083 can mitigate myofiber pathology and intracellular TDP-43 mislocalization in the VCP^R155H/R155H^ mice.

### The effect of CB-5083 treatment on biomarker expressions in VCP^R155H/R155H^ mice

**Fig. 4:**
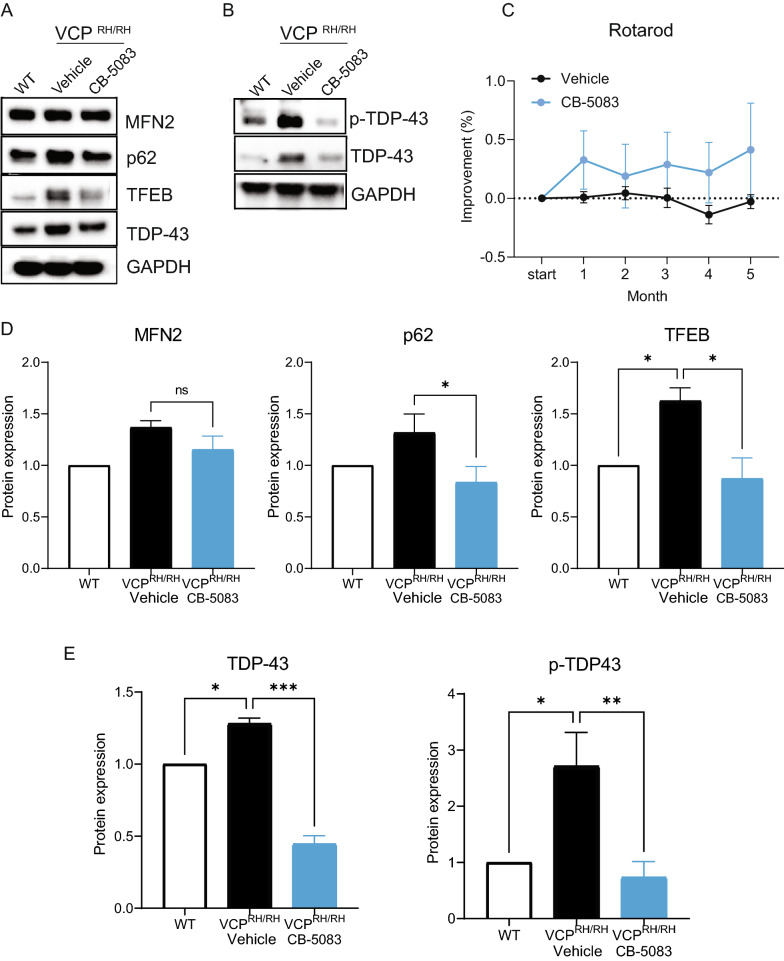
Biomarker analysis of VCP^R155H/R155H^ mice treated with CB-5083. **A** Quadriceps lysates of WT, and vehicle and CB-5083-treated VCP^R155H/R155H^ mice (n = 3–5 in each group) were subjected to Western blot analysis and immunoblotted with antibodies against major disease biomarkers including MFN2, p62, TFEB, and TDP-43. GAPDH was used as a loading control. p62, TFEB, and TDP-43 levels were diminished in VCP^R155H/R155H^ mice upon CB-5083 treatment. **B** Western blots of quadriceps lysates, immunoblotted with antibody against TDP-43 and phosphorylated TDP-43 (p-TDP-43). P-TDP-43 was diminished in VCP^R155H/R155H^ muscles upon CB-5083 treatment. **C** Rotarod analysis for VCP^R155H/R155H^ mice treated with CB-5083 (n = 8) compared to vehicle treatment (n = 7) showed differences, however were not significant, **D** Densitometric analysis of Western blots shown in (**A**, **B**). Western blot was repeated three times. Statistical analysis was performed by one-way ANOVA followed by Fisher’s LSD test *P < 0.05

Previous studies have revealed several pathological biomarkers that are the hallmark of VCP disease: (1) Elevated TDP-43 levels and its translocation from the nucleus to the cytoplasm [32–36], and elevation of phosphorylated TDP-43 level, (2) Components of autophagy pathways including p62 which are elevated, suggesting an induced autophagic pathway in the disease condition, (3) Elevation of transcription factor EB (TFEB) involved in lysosomal damage response pathways in the VCP^R155H/+^ and VCP knockout mice [37], indicating that lysosome homeostasis is disrupted in the conditions of both VCP deficiency and hyperactivity. Analysis of the drosophila model harboring VCP mutations revealed that VCP is necessary for the degradation of mitofusin proteins. Treating VCP mutant flies with VCP inhibitors, NMS-873 and ML240 resulted in an improvement of mitochondrial pathology and an elevation in levels of mitofusin proteins [16, 35]. To evaluate the effect of CB-5083 on the expression levels of the above-mentioned biomarkers, we compared the protein expression levels in WT, vehicle-treated and CB-5083-treated VCP^R155H/R155H^ mice. We found that TDP-43 levels were significantly increased in the VCP^R155H/R155H^ mouse quadriceps muscles compared to WT littermate controls, and this increase was mitigated by the 5-month treatment with CB-5083 (Fig. 4A, B). Elevation of p62 and TFEB in VCP^R155H/R155H^ muscles were normalized to WT-equivalent levels upon CB-5083 treatment (Fig. 4A, B, D). In contrast to the findings from previous drosophila studies [16, 35], we did not detect significant differences in MFN2 levels in the VCP^R155H/R155H^ mice compared to WT mice, and CB-5083 did not modulate MFN2 levels (Fig. 4A). Importantly, the elevated TDP-43 phosphorylation in VCP^R155H/R155H^ mice was also significantly reduced with CB-5083 treatment (Fig. 4B, D). Altogether, our results suggest that CB-5083 treatment alleviates the major disease biomarkers involved in multiple cellular pathways in the VCP^R155H/R155H^ mice and may have translational potential.

The chronic CB-5083 treatment with the dose of 15 mg/kg for 5 months did not appear to have any adverse effect on deteriorating motor function (Fig. 4C). We did not observe significant functional changes in Rotarod behavior in CB-5083-treated VCP^R155H/R155H^ mice (n = 8) compared to vehicle treatment (n = 7) (Fig. 4C).

### CB-5083 did not affect CNS pathology of VCP disease

**Fig. 5:**
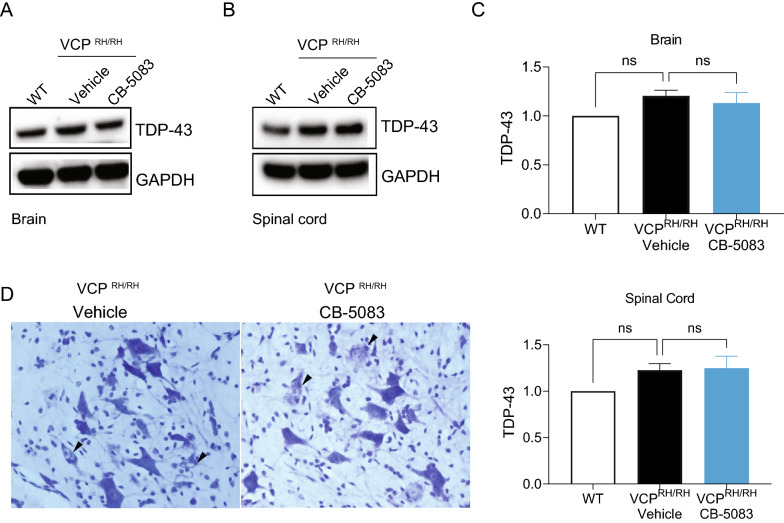
Effects of CB-5083 in CNS of VCP^R155H/R155H^ mice. Whole-brain lysates (**A**) and spinal cords lysates (**B**) from WT, vehicle-treated and CB-5083-treated VCP^R155H/R155H^ mice (n = 3 per group) were subjected to immunoblotting using antibodies against TDP-43 and GAPDH. The TDP-43 level was not altered upon CB-5083 treatment. **C** Quantification of Western blots shown in (**A**, **B**). **D** Motor neuron lesions were identified in spinal cords of VCP^R155H/R155H^ mice treated with either CB-5083 or vehicle. Arrowhead points to the injured neurons. Western blot was repeated three times. Statistical analysis was performed by one-way ANOVA followed by Fisher’s LSD test *P < 0.05

Patients with VCP disease also present with CNS pathology [24, 31, 38]. The major pathological signature includes neuron degeneration, and ubiquitin- and TDP-43-positive cytoplasmic aggregates in dystrophic neurites [38, 39]. In rodent models of VCP disease, cytoplasmic accumulation of TDP-43 is observed widely across brain regions [24, 30, 34, 38]. Even though CB-5083 bioavailability in the brains is one-third of the levels in the circulating blood [40], we wanted to assess if CB-5083 treatment for 5 months could modulate these important disease phenotypes in VCP^R155H/R155H^ mice. TDP-43 expression in the vehicle-treated VCP^R155H/R155H^ mice at 7 months was not significantly altered in the brain and the spinal cord compared to the wildtype controls (Fig. 5A–C). These results are consistent with previous findings showing that TDP-43 pathology in the brain appears later and is milder compared to the pathological onset in muscles [24, 30, 34, 38]. Additionally only 30% of patients develop frontotemporal dementia and approximately 10% develop amyotropic lateral sclerosis. We found that TDP-43 expression was reduced in the brain but not in the spinal cord after 5 months of CB-5083 treatment, however this difference was not statistically significant (Fig. 5A–C).

Upon characterization of the spinal cord pathology in the VCP^R155H/R155H^ mice by Nissl staining, we identified lesions in the mouse spinal cords. In both vehicle and CB-5083-treated spinal cords, atrophy, swelling, and various degrees of neuronal cell injury were observed (Fig. 5D), reminiscent of previous pathological findings in VCP^R155H/R155H^ mice [24, 38].

### The effect of CB-5083 on biomarker expression in aging heterozygous VCP^R155H/+^ mice

**Fig. 6:**
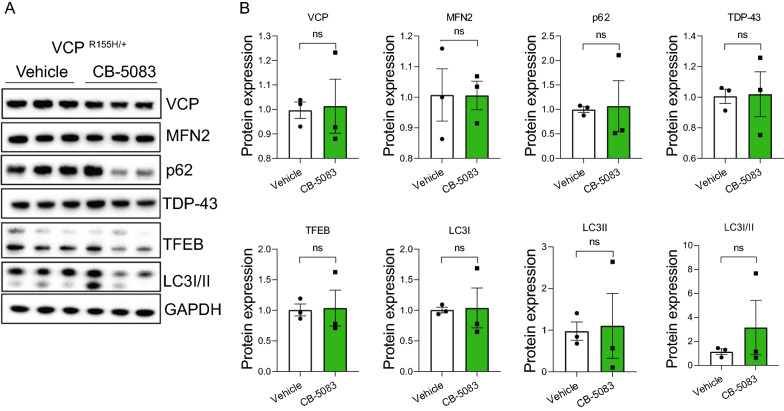
Analysis of aged VCP^R155H/+^ mice with CB-5083 treatment. VCP^R155H/+^ mice were treated with CB-5083 for 6 months starting from 12 months of age. **A** Muscle lysates were generated from VCP^R155H/+^ mice treated with and without CB-5083 (n = 3 per group) and subjected to immunoblotting using anti-VCP, MFN2, p62, TDP-43, TFEB, and LC3I/II antibodies. GAPDH was used as a loading control. **B** Quantification of Western blot analysis shown in (**A**). A trend of diminution of biomarker expression was observed for VCP^R155H/+^ mice treated with CB-5083, especially in p62, TFEB, and LC3I/II. Western blot was repeated three times. Statistical analysis was performed by one-way ANOVA followed by Fisher’s LSD test

Additionally, we tested whether chronic CB-5083 treatment has an effect in aged VCP^R155H/+^ mice. We treated 12-month-old VCP^R155H/+^ mice with 15 mg/kg CB-5083 for 6 months. Despite the variation of biomarker expression with CB-5083 treatment, we found a slight trend towards reduced p62, LC3I/II, and TFEB levels (Fig. 6). These findings were similar to what was observed with younger CB-5083-treated VCP^R155H/R155H^ mice (Fig. 4), with the exception that we did not observe a reduction in TDP-43 levels (Fig. 6).

### Chronic CB-5083 treatment does not have permanent suppressive effects on retinal function

**Fig. 7:**
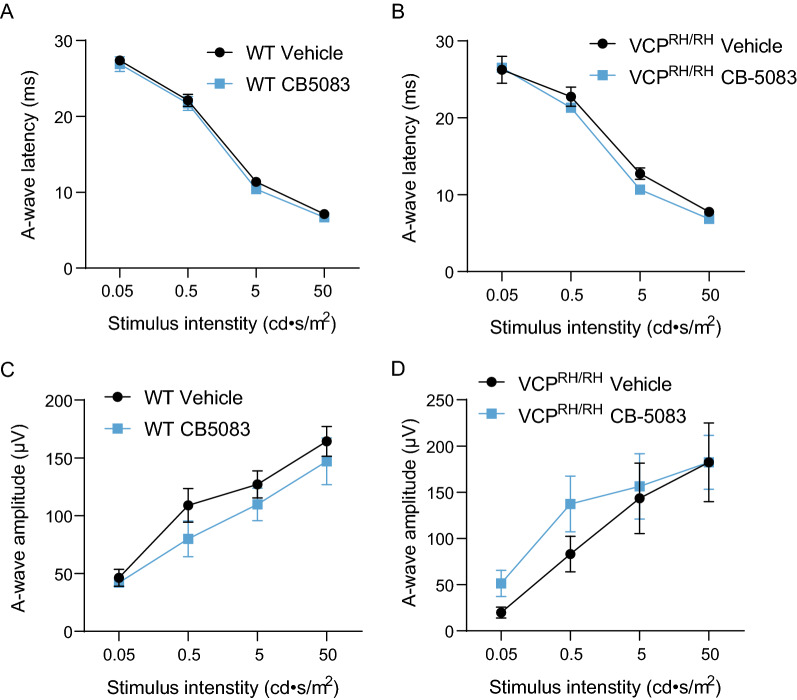
Electroretinographic (ERG) analysis of the effects of CB-5083 on retinal function. **A**, **B** ERG analysis of photoresponse (ERG A-wave component) latency showed no differences between vehicle and CB-5083 treatment in both WT and VCP^R155H/R155H^ mice. **D**, **E** ERG analysis of photoresponse (ERG A-wave component) amplitude showed no differences between vehicle and CB-5083 treatment in both WT and VCP^R155H/R155H^ mice. Statistical analysis was performed by one-way ANOVA followed by Fisher’s LSD test

During the drug development pipeline, a battery of tests was performed to assess CB-5083’s potential off-target effects. In a panel of 229 ATPases, CB-5083 was found to have no activity of concern for clinical development in oncology. However, visual disturbances during the Phase 1 trials in oncology were reported. Therefore, further characterization of CB-5083 was conducted and an inhibition constant of 33 nM was identified for phosphodiesterase-6 (PDE6). PDE6 is an essential enzyme for photoreceptor function, converting the photons’ energy from visible light into a neural signal; and PDE6 is a known off-target liability for certain PDE5 inhibitors used clinically to treat erectile dysfunction [18, 41, 42]. A previous phase I clinical trial conducted by Cleave Therapeutics evaluated the safety of CB-5083 in patients with advanced solid tumors or relapsed refractory multiple myeloma. In this trial, patients experienced adverse visual events including photophobia, photopsia, and dyschromatopsia, contributing to the cessation of the Phase I trial during the dose-escalation phase. To evaluate the potential for adverse visual effects in the context of VCP disease, we performed electroretinographic (ERG) analysis in VCP^R155H/R155H^ mice after a 5-month treatment with CB-5083 to evaluate if the drug has permanent deleterious effects on retinal function. To exclude the effects of acute PDE6 suppression on the ERG recording, we allowed a 24-h washout after the last CB-5083 administration. We found no drug effects on photoresponse kinetics or amplitude (Fig. 7A–D), indicating that CB-5083 is well tolerated and the suppressive effect on PDE6 and retinal function is reversible. Our earlier investigation in WT and VCP^R155H/+^ mice led to the same conclusion [40].

## Discussion

Previous studies have shown that both VCP mutant homohexamers and VCP heterohexamers containing both WT and VCP mutant monomers have elevated AAA ATPase enzyme activity [43], making them susceptible to inhibition by CB-5083. In this study, we evaluated the safety and efficacy of the competitive and reversible VCP inhibitor CB-5083 in patient-derived myoblast cells and the VCP disease mouse model. We found that the mice tolerated chronic CB-5083 treatment with no detectable adverse effects. Importantly, CB-5083 did not lead to long-term alteration in retinal function. Our comprehensive muscle pathology analyses indicated that the characteristic phenotypes in VCP^R155H/R155H^ mice were prominently mitigated by CB-5083 treatment. Overall, our study indicates that CB-5083 could be a safe and efficacious drug to treat muscle pathology in VCP disease, supporting the treatment of patients with VCP disease in a clinical setting.

This study is the first one to show the potential of a VCP inhibitor to mitigate muscle pathology in a mammalian model of VCP disease. CB-5083 improved myofiber structure in VCP^R155H/R155H^ mice, reduced cytoplasmic TDP-43 accumulation, and improved abnormal biomarker expression. We found a similar trend in the older VCP^R155H/+^ mice. However, in the aged VCP^R155H/+^ mice, TDP-43 level is less responsive to CB-5083 treatment, perhaps due to aged VCP^R155H/+^ mice harboring more advanced pathology. It is also conceivable that aging renders clearing out aggregates more challenging. Our results further strengthen the hypothesis that *VCP* gain-of-function variants are causal for the disease pathogenesis [15, 18, 19] and that inhibition of VCP activity has translational potential, not only for cancer but also for *VCP*-associated myopathy. Previously reported studies have shown that VCP homohexamers with pathogenic VCP variants have increased unfoldase activity, which can be inhibited back to the wildtype levels [17]. In our rapidly progressing VCP disease model, VCP^R155H/R155H^ mice, the endogenous VCP hexamer complexes are composed entirely of mutant monomers. Based on our findings that VCP inhibitor CB-5083 is beneficial in improving muscle pathology, we propose that correcting the enzyme hyperactivity could be therapeutic in patients. Additionally, we interpret the lack of a significant effect of CB-5083 in the spinal cord or the brain as due to low CNS bioavailability [40]. Therefore, it will be valuable to examine the effect of future VCP inhibitors that have improved CNS penetrance.

Previous reports studying VCP inhibition have demonstrated that VCP has a multifaceted physiological role, including the regulation of autophagy pathways [9]. We found that CB-5083 can influence autophagy protein levels in patient-derived myoblasts and in the muscles of VCP disease mouse models. Specifically, when myoblasts were treated acutely with increasing doses of CB-5083 for 5 days, we observed a consistent upregulation of autophagic markers, in line with the previous findings that VCP knockdown stimulates autophagy [10, 35]. These data suggest that both the expression level and the enzymatic function of VCP are required for regulating autophagy pathways. We hypothesize that the 5-month-long chronic treatment of mice with CB-5083 had a physiological beneficial effect in alleviating protein buildup burden and thus decreased the physiological needs for autophagy induction in vivo.

MFN2 was significantly upregulated in the myoblast culture with CB-5083 treatment similar to previous findings [18]. These results indicate that CB-5083 is effective as an inhibitor of VCP, which is known to negatively regulate the turnover of MFN2. Interestingly, MFN2 was not altered in the muscles of the mutant mice compared to WT mice, and we did not see significant changes with CB-5083 treatment. Perhaps the heterogeneous populations of cells in the muscle tissue lysates cannot reflect in-vivo homeostatic mechanisms.

Finally, our analysis of retinal function in WT and VCP^R155H/R155H^ mice after chronic CB-5083 treatment provides further evidence that CB-5083’s inhibition of PDE6 in retinal function is reversible. No retinal abnormality was observed in the current study when mice were allowed a 24-h drug wash-out before ERG recording. In our previous study characterizing the acute effect of CB-5083 in retinal function, single administration of CB-5083 can lead to dose-dependent ERG signal deterioration consistent with the observed PDE6 inhibitory activity by CB-5083. However, also in the study that solely focused on CB-5083’s effects in the eye, CB-5083 did not cause any signs of permanent retinal anomalies after a 6-month follow-up in aged VCP model mice [40]. Altogether, these data support that the side effects of CB-5083 are transient and do not cause permanent ocular toxicity. In summary, our study indicates that CB-5083 is a safe and potentially efficacious treatment for muscle pathology in VCP disease.

## Material and methods

### VCP R155H knock-in mice

All animal experiments were approved by the Institutional Animal Care and Use Committees (IACUC) at the University of California, Irvine. Mice were maintained under regular housing conditions with ad libitum access to food and drink in a pathogen-free facility. The immunohistochemistry, Western blot, and retinal electrophysiology procedures were carried out using 4-month-old to 12-month-old mice of both sexes. Genotypes were determined using PCR from tail or ear punches (Transnetyx, Inc., Cordova, TN). The VCP R155H mice were generated at InGenious Targeting Laboratory, Inc. (Ronkonkoma, NY), and have been backcrossed to C57BL/6J for at least 10 generations [23, 26]. VCP R155H mice are also available through the Jackson Laboratory (Bar Harbor, ME) (Stock #021968).

### Primary myoblast culture

Mutant cells with heterozygous p.R155H and p.R93C mutations were obtained from the EuroBioBank (Munich, Germany). These mutant cells were generated from the muscle biopsies of patients showing typical clinical phenotype and histological findings of VCP disease. Control myoblasts were obtained from EuroBioBank and were generated from age-matched control subjects without any pathological findings. Cells were grown on plates coated with Gelatin (Stemcell #07903) in Skeletal Growth Medium (Promo Cell C23060) at 37 °C and 5% CO_2_ [44]. The identity and purity of the cultures were confirmed by morphological analyses and immunocytochemical stainings.

### CB-5083 treatment of primary myoblasts

CB-5083 was reconstituted in DMSO (MP Biomedical #196055) at the concentration of 5 mg/ml and stored at − 20 °C as stock. During cell treatment, 5 mg/ml CB-5083 was serially diluted in Skeletal Growth Medium (Promo Cell C23060) to 50–300 nM concentrations and added to myoblasts (at ~ 70% confluency). Treated myoblasts were incubated for 4–5 days, followed by whole-cell lysis.

### Protein lysates and Western blot

Preparation of protein lysates and Western blot analyses were performed as previously described [45]. Primary cells were washed with phosphate buffered saline (PBS) and harvested in RIPA buffer (ThermoFisher) with protease inhibitor cocktail, incubated on ice for at least 10 min, and centrifuged at > 20,000*g* for 20 min at 4 °C. The supernatant was collected as the total protein lysate. Approximately 30 mg of frozen muscle was homogenized in either RIPA or lysis buffer (50 mM Tris-HCl, 150 mM NaCl, 1% Triton-X) using a Dounce homogenizer (Wheaton Dounce Tissue Grinder, Catalog #357538) with 30–40 strokes. The homogenate was then passed through a 25-gauge syringe 15 times to shear tissue trunks, rotated at 4 °C for ~ 2 h, and centrifuged at > 20,000*g* at 4 °C for 30 min. The supernatant was collected as total muscle lysate. Nuclear/cytoplasmic fractionation was conducted based on the manufacturer’s protocol (ThermoFisher 78833). Protein lysates were subjected to Western blot and immunoblotted with primary antibodies against GAPDH (1:10,000, Abcam#181602), TDP-43 (1:3000, Abcam#190963), LC3I/II (1:1500, Abcam#192890), p62 (1/5000 Abcam#56416), TFEB (1:3000, Bethyl Laboratory303-673A), MFN2 (1:2000, Abcam#56889), S409/410 p-TDP-43 (1:2500, Cosmo Bio USA: CAC-TIP-PTD-M01). Goat anti-mouse horseradish peroxidase (HRP) or goat anti-rabbit HRP (1:5000) were used as secondary antibodies. For the experiments conducted with VCP^R155H/R155H^ mice, 3 mice per group were analyzed to assess biomarker expression. For the experiments conducted with VCP^R155H/+^ mice, five pairs of mice were analyzed to assess biomarker expression. Western blot was repeated at least twice.

### MTT assay

The 3-(4,5-dimethylthiazol-2-yl)-2,5-diphenyl-2H-tetrazolium bromide **(**MTT) assay (SigmaM 5655) was used to assess the cell survival rate upon CB-5083 treatment. Myoblasts were plated at the density of 1 × 10^4^ cells per well in the 96-well plate, and treated with dimethylsulfoxide (DMSO) vehicle control; or 50 nM, 75 nM, 100 nM, 150 nM, 200 nM or 300 nM of CB-5083 through serial dilution. Cells were treated for 5 days. When ready, 10 µl of MTT solution was added to 100 µl of cell medium in each well. Cells were incubated at 37 °C for 3 h, followed by the addition of 100 µl MTT solubilization solution in each well. Absorbances were measured at 570 nm for signals, and 690 nm for background.

### Immunohistochemistry

Immunohistochemical analyses were performed as described [45]. Primary myoblasts were cultured in 8-well chamber slides coated with gelatin. Immunohistochemistry was conducted for myoblasts and muscle sections. Muscles from mice were harvested freshly frozen and subjected to cryosectioning, followed by either immunofluorescence staining or H&E staining. Spinal cords from mice were fixed with 4% paraformaldehyde (PFA) and 30% sucrose and subjected to cryosectioning and Nissl staining. Muscle sections or myoblasts were fixed with 4% PFA for 10 min, 0.2% Triton-X permeabilization for 10 min, 10% donkey serum in tris-buffered saline with 0.1% Triton-X (TBST) blocking for 1 h, anti-TDP-43 rabbit polyclonal antibody (1:200), anti-Desmin rabbit antibody (1:200, Abcam#15200) at 4 °C overnight and followed by goat anti-rabbit Alexa Fluor-488 secondary antibodies (1:500) at room temperature for 1 h. All images were acquired with Keyence with identical configurations for all samples in the same experiment.

### Serum biochemistry

Blood samples were collected from mice treated with CB-5083 or vehicle for 5 months. Blood samples were collected under anesthesia using isoflurane. Blood samples were drawn from the heart and transferred into serum tubes (Minicollect ref 450472). Clinical biochemistry testing was obtained from IDEXX Laboratories. Parameters evaluated included contents of alanine aminotransferase, aspartate aminotransferase, alkaline phosphatase, gamma-glutamyl transferase, blood urea nitrogen, creatine kinase, creatinine, total protein, albumin, and total bilirubin.

### In vitro and in vivo treatment with CB-5083

For in vitro treatment with CB-5083, solid CB-5083 was solubilized in DMSO at the concentration of 5 mg/ml. Variable dosages of CB-5083 were used for cell treatments with DMSO as vehicle control. For in vivo treatment with CB-5083, solid CB-5083 was solubilized in methylcellulose at the concentration of 3 mg/ml. Methylcellulose solution was used as vehicle control for in vivo treatment. Mice were treated with daily oral gavage. For maximum tolerated dosage analysis, 12-month-old VCP^R155H/+^ were treated daily with CB-5083 at doses of 15 mg/kg or 25 mg/kg for 2 weeks. Body weights were monitored weekly. For chronic treatment with CB-5083 to assess safety and efficacy, VCP^R155H/R155H^ mice were treated daily for 5 months starting from 2 months of age, and the VCP^R155H/+^ mice were treated from 12 months of age for 6 months.

### Electroretinography

Mice were anesthetized by an intraperitoneal injection of ketamine (100 mg/kg, KetaVed, Bioniche Teoranta, Inverin Co, Galway, Ireland) and xylazine (10 mg/kg, Rompun, Bayer, Shawnee Mission, KS), and their pupils were dilated with 1% tropicamide (Tropicamide Ophthalmic Solution, Akorn, Lake Forest, IL). Thereafter, the corneas were moistened using 0.3% hypromellose gel (GenTeal, Alcon, Fort Worth, TX) which also secured electrical conductivity during electroretinography (ERG) recording. The ERG was performed as previously described [46] using a Diagnosys Celeris rodent ERG device (Diagnosys LLC, Lowell, MA). Briefly, mice were dark-adapted overnight, and all handling before ERG was done under dim red light (> 600 nm). After electrodes were attached, three more minutes were allowed to fully dark-adapt the animal before stimulation. Stimulation was performed using a green LED (peak emission at ∼ 544 nm, bandwidth ∼ 160 nm) and an ascending 1-log step light intensity series between 0.0005–50 cd s/m^2^.

### Image quantification

Fiji Image J was used to quantify the percentage of myofibers with TDP-43 cytoplasmic signal. We first counted how many total myofibers were in one image, then we counted how many myofibers had TDP-43 positive signals in the cytoplasms of the myofibers. Percentage of the myofibers with TDP-43 cytoplasmic signal was calculated and compared across WT, Vehicle, and CB-5083-treated VCP^R155H/R155H^ mice (n = 4 for CB-5083 treatment, n = 3 for vehicle treatment, and n = 4 for WT). To quantify the number of the centralized nuclei from the H&E staining, at least 300 myofibers were manually counted for each mouse, and the numbers of the centralized nuclei were recorded (n = 3 mice for each group). We identified muscle sections from similar regions of the quadriceps muscles from different mice by (1) selecting muscle sections with similar cross-sectional area, and (2) counting the number of sections from the beginning. The data were presented as the percentage of the centralized nuclei.

### Statistical analysis

Data are presented as mean ± SEM. One-way ANOVA test followed by Fisher’s LSD was used for Western blot densitometry analyses, image analyses, and ERG analyses. For blood toxicology and liver enzyme analyses, Mann–Whitney U-test was used. Simple main effects were calculated for significant main effects of factors. The level of statistical significance was set at *P* < 0.05.

## Supplementary Information

**Additional file 1: Figure S1.** Characterization of the VCP disease patient-derived myoblasts. (A) Quantification of the Western blot analysis of cytoplasmic mitofusin, cytoplasmic and nuclear TDP-43 and cytoplasmic p62, shown in Fig. 1B. Cytoplasmic p62 was significantly elevated in the patient myoblasts. (B) Quantification of the Western blot analysis of cytoplasmic LC3 I/II levels reveals that cytoplasmic LC3 I level was increased in the patient myoblasts. (C) Quantification of the percentage of the myoblasts with TDP-43 cytoplasmic expansion. (D) Patient-derived myoblasts were treated with 75 nM CB-5083 for 5 days, followed by nuclear and cytoplasmic fraction analysis of TDP-43 and Ubiquitin. Ubiquitin level was not changed. (E) Quantification of western blot shown in (D) revealed that TDP-43 cytoplasmic level was not changed. The experiments were performed in two patient-derived and control myoblasts. The Western blot was repeated at least twice. Statistical analysis was performed by one-way ANOVA followed by Fisher’s LSD test.

## Data Availability

All data generated or analyzed during this study are included in this article (and its additional files).

## References

[1] Virginia K. Inclusion body myopathy with paget disease of bone and/or frontotemporal dementia. GeneReviews®. 2007. https://www.ncbi.nlm.nih.gov/books/NBK1476/.

[2] Kimonis VE, Kovach MJ, Waggoner B, Leal S, Salam A, Rimer L (2000). Clinical and molecular studies in a unique family with autosomal dominant limb-girdle muscular dystrophy and Paget disease of bone. Genet Med.

[3] Al-Obeidi E, Al-Tahan S, Surampalli A, Goyal N, Wang AK, Hermann A (2018). Genotype-phenotype study in patients with valosin-containing protein mutations associated with multisystem proteinopathy. Clin Genet.

[4] Mehta SG, Khare M, Ramani R, Watts GDJ, Simon M, Osann KE (2013). Genotype-phenotype studies of VCP-associated inclusion body myopathy with Paget disease of bone and/or frontotemporal dementia. Clin Genet.

[5] Farpour F, Tehranzadeh J, Donkervoort S, Smith C, Martin B, Vanjara P (2012). Radiological features of Paget disease of bone associated with VCP myopathy. Skelet Radiol.

[6] Kimonis VE, Fulchiero E, Vesa J, Watts G (2008). VCP disease associated with myopathy, Paget disease of bone and frontotemporal dementia: review of a unique disorder. Biochim Biophys Acta - Mol Basis Dis.

[7] Ralston SH (2020). Bisphosphonates in the management of Paget’s disease. Bone.

[8] Watts GDJ, Wymer J, Kovach MJ, Mehta SG, Mumm S, Darvish D (2004). Inclusion body myopathy associated with Paget disease of bone and frontotemporal dementia is caused by mutant valosin-containing protein. Nat Genet.

[9] Meyer H, Weihl CC (2014). The VCP/p97 system at a glance: connecting cellular function to disease pathogenesis. J Cell Sci.

[10] Ju JS, Weihl CC (2010). p97/VCP at the intersection of the autophagy and the ubiquitin proteasome system. Autophagy.

[11] Ju JS, Weihl CC (2010). Inclusion body myopathy, Paget’s disease of the bone and fronto-temporal dementia: a disorder of autophagy. Hum Mol Genet.

[12] Nalbandian A, Llewellyn KJ, Gomez A, Walker N, Su H, Dunnigan A (2015). In vitro studies in VCP-associated multisystem proteinopathy suggest altered mitochondrial bioenergetics. Mitochondrion.

[13] Fernández-Sáiz V, Buchberger A (2010). Imbalances in p97 co-factor interactions in human proteinopathy. EMBO Rep.

[14] Niwa H, Ewens CA, Tsang C, Yeung HO, Zhang X, Freemont PS (2012). The role of the N-domain in the atpase activity of the mammalian AAA ATPase p97/VCP. J Biol Chem.

[15] Halawani D, LeBlanc AC, Rouiller I, Michnick SW, Servant MJ, Latterich M (2009). Hereditary inclusion body myopathy-linked p97/VCP mutations in the NH2 domain and the D1 ring modulate p97/VCP ATPase activity and D2 ring conformation. Mol Cell Biol.

[16] Manno A, Noguchi M, Fukushi J, Motohashi Y, Kakizuka A (2010). Enhanced ATPase activities as a primary defect of mutant valosin-containing proteins that cause inclusion body myopathy associated with Paget disease of bone and frontotemporal dementia. Genes Cells.

[17] Nalbandian A, Llewellyn KJ, Nguyen C, Monuki ES, Kimonis VE (2015). Targeted excision of VCP R155H mutation by Cre-LoxP technology as a promising therapeutic strategy for valosin-containing protein disease. Hum Gene Ther Methods.

[18] Zhang T, Mishra P, Hay BA, Chan D, Guo M (2017). Valosin-containing protein (VCP/p97) inhibitors relieve mitofusin-dependent mitochondrial defects due to VCP disease mutants. Elife.

[19] Blythe EE, Olson KC, Chau V, Deshaies RJ (2017). Ubiquitin- A nd ATP-dependent unfoldase activity of P97/VCP•NPLOC4•UFD1L is enhanced by a mutation that causes multisystem proteinopathy. Proc Natl Acad Sci USA.

[20] Chou TF, Bulfer SL, Weihl CC, Li K, Lis LG, Walters MA (2014). Specific inhibition of p97/VCP ATPase and kinetic analysis demonstrate interaction between D1 and D2 ATPase domains. J Mol Biol.

[21] Zhou HJ, Wang J, Yao B, Wong S, Djakovic S, Kumar B (2015). Discovery of a First-in-class, potent, selective, and orally bioavailable inhibitor of the p97 AAA ATPase (CB-5083). J Med Chem.

[22] Magnaghi P, D’Alessio R, Valsasina B, Avanzi N, Rizzi S, Asa D (2013). Covalent and allosteric inhibitors of the ATPase VCP/p97 induce cancer cell death. Nat Chem Biol.

[23] Le Moigne R, Aftab BT, Djakovic S, Dhimolea E, Valle E, Murnane M (2017). The p97 inhibitor CB-5083 is a unique disrupter of protein homeostasis in models of multiple myeloma. Mol Cancer Ther.

[24] Nalbandian A, Llewellyn KJ, Kitazawa M, Yin HZ, Badadani M, Khanlou N (2012). The Homozygote VCPR155H/R155H mouse model exhibits accelerated human VCP-associated disease pathology. PLoS ONE.

[25] Eric D, David F, Angele N, Matthew G, Veeral K, Prachi R (2014). Disease-specific induced pluripotent stem cell modeling: insights into the pathophysiology of valosin containing protein (VCP) disease. J Stem Cell Res Ther.

[26] Parzych K, Saavedra-García P, Valbuena GN, Al-Sadah HA, Robinson ME, Penfold L (2019). The coordinated action of VCP/p97 and GCN2 regulates cancer cell metabolism and proteostasis during nutrient limitation. Oncogene.

[27] Anderson DJ, Le Moigne R, Djakovic S, Kumar B, Rice J, Wong S (2015). Targeting the AAA ATPase p97 as an approach to treat cancer through disruption of protein homeostasis. Cancer Cell.

[28] Hill SM, Wrobel L, Ashkenazi A, Fernandez-Estevez M, Tan K, Bürli RW (2021). VCP/p97 regulates Beclin-1-dependent autophagy initiation. Nat Chem Biol.

[29] Badadani M, Nalbandian A, Watts GD, Vesa J, Kitazawa M, Su H (2010). VCP associated inclusion body myopathy and paget disease of bone knock-in mouse model exhibits tissue pathology typical of human disease. PLoS ONE.

[30] Nalbandian A, Llewellyn KJ, Badadani M, Yin Z, Nguyen C, Katheria V (2013). A progressive translational mouse model of human VCP disease: the VCP R155H/+ mouse. Muscle Nerve.

[31] Llewellyn KJ, Nalbandian A, Jung KM, Nguyen C, Avanesian A, Mozaffar T (2014). Lipid-enriched diet rescues lethality and slows down progression in a murine model of VCP-associated disease. Hum Mol Genet.

[32] Ahmed M, MacHado PM, Miller A, Spicer C, Herbelin L, He J (2016). Targeting protein homeostasis in sporadic inclusion body myositis. Sci Transl Med.

[33] Vogler TO, Wheeler JR, Nguyen ED, Hughes MP, Britson KA, Lester E (2018). TDP-43 and RNA form amyloid-like myo-granules in regenerating muscle. Nature.

[34] Custer SK, Neumann M, Lu H, Wright AC, Taylor JP (2010). Transgenic mice expressing mutant forms VCP/p97 recapitulate the full spectrum of IBMPFD including degeneration in muscle, brain and bone. Hum Mol Genet.

[35] Ju JS, Fuentealba RA, Miller SE, Jackson E, Piwnica-Worms D, Baloh RH (2009). Valosin-containing protein (VCP) is required for autophagy and is disrupted in VCP disease. J Cell Biol.

[36] Weihl CC, Temiz P, Miller SE, Watts G, Smith C, Forman M (2008). TDP-43 accumulation in inclusion body myopathy muscle suggests a common pathogenic mechanism with frontotemporal dementia. J Neurol Neurosurg Psychiatry.

[37] Arhzaouy K, Papadopoulos C, Schulze N, Pittman SK, Meyer H, Weihl CC (2019). VCP maintains lysosomal homeostasis and TFEB activity in differentiated skeletal muscle. Autophagy.

[38] Yin HZ, Nalbandian A, Hsu CI, Li S, Llewellyn KJ, Mozaffar T (2012). Slow development of ALS-like spinal cord pathology in mutant valosin-containing protein gene knock-in mice. Cell Death Dis.

[39] Neumann M, Mackenzie IR, Cairns NJ, Boyer PJ, Markesbery WR, Smith CD (2007). TDP-43 in the ubiquitin pathology of frontotemporal dementia with VCP gene mutations. J Neuropathol Exp Neurol.

[40] Leinonen H, Cheng C, Pitkänen M, Sander CL, Zhang J, Saeid S (2021). A p97/valosin-containing protein inhibitor drug CB-5083 has a potent but reversible off-target effect on phosphodiesterase-6. J Pharmacol Exp Ther.

[41] Zhang X, Feng Q, Cote RH (2005). Efficacy and selectivity of phosphodiesterase-targeted drugs in inhibiting photoreceptor phosphodiesterase (PDE6) in retinal photoreceptors. Investig Ophthalmol Vis Sci.

[42] Hatzimouratidis K, Salonia A, Adaikan G, Buvat J, Carrier S, El-Meliegy A (2016). Pharmacotherapy for erectile dysfunction: recommendations from the fourth international consultation for sexual medicine (ICSM 2015). J Sex Med.

[43] Blythe EE, Gates SN, Deshaies RJ, Martin A (2019). Multisystem proteinopathy mutations in VCP/p97 increase NPLOC4·UFD1L binding and substrate processing. Structure.

[44] Mora M, Bragato C, Gibertini S, Zanotti S, Curcio M, Canioni E (2017). Biobank of cells, tissues and DNA from patients with neuromuscular diseases: an indispensable link between clinical centers and the scientific community. Open J Bioresour.

[45] Cheng C, Deng PY, Ikeuchi Y, Yuede C, Li D, Rensing N (2018). Characterization of a mouse model of Börjeson–Forssman–Lehmann syndrome. Cell Rep.

[46] Orban T, Leinonen H, Getter T, Dong Z, Sun W, Gao S (2018). A combination of G protein-coupled receptor modulators protects photoreceptors from degeneration. J Pharmacol Exp Ther.

