# Supplementary figures and images for: VCP/p97 inhibitor CB-5083 modulates muscle pathology in a mouse model of VCP inclusion body myopathy
Source: J Transl Med. 2022 Jan 8;20:21. doi: 10.1186/s12967-021-03186-6 (PMC8742393; doi:10.1186/s12967-021-03186-6)

A

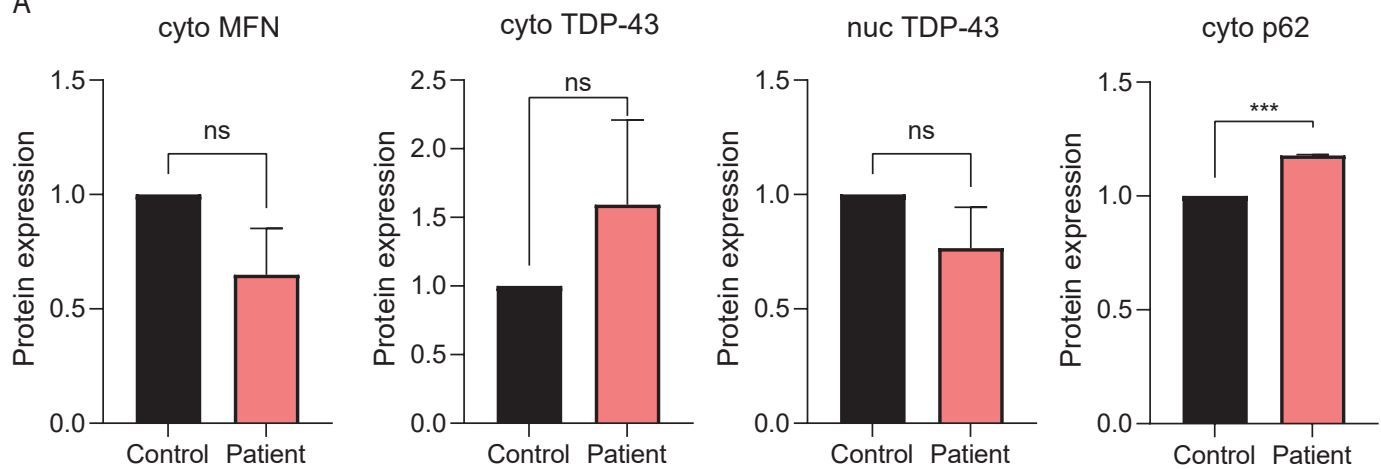

B

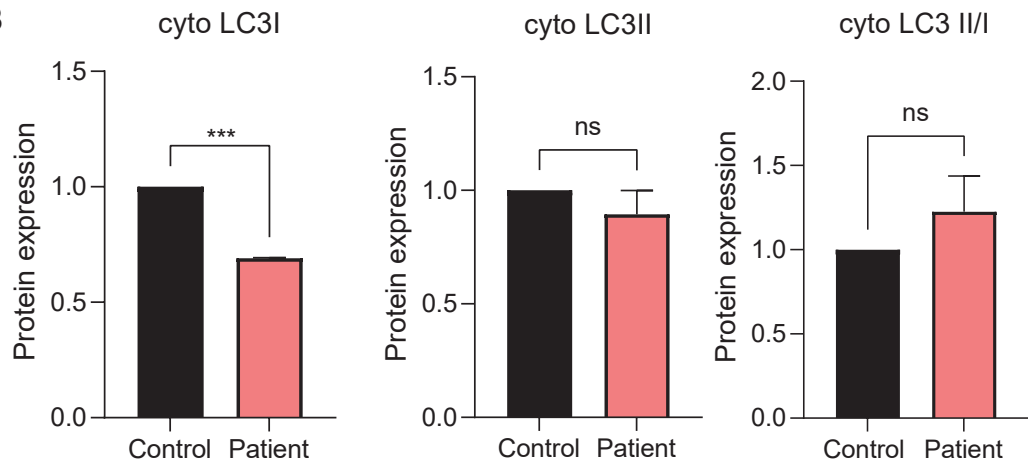

C

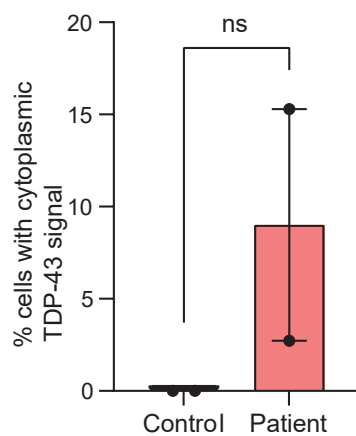

D

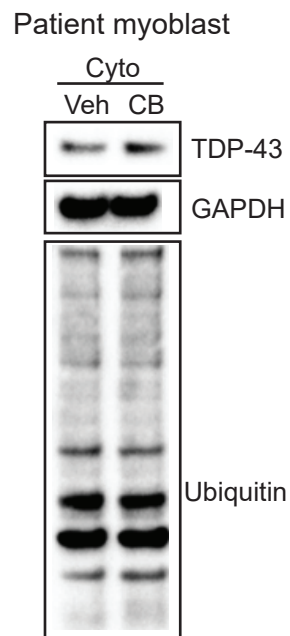

E

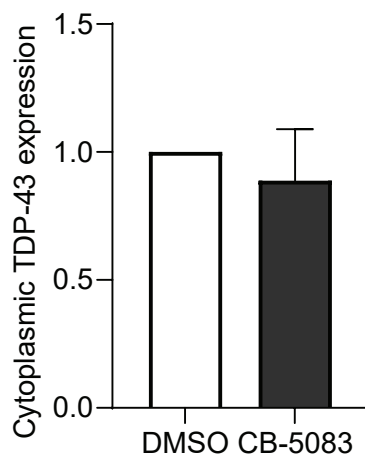

Supplement: Supplementary file 1 — Additional file 1: Figure S1. Characterization of the VCP disease patient-derived myoblasts. (A) Quantification of the Western blot analysis of cytoplasmic mitofusin, cytoplasmic and nuclear TDP-43 and cytoplasmic p62, shown in Fig. 1B. Cytoplasmic p62 was significantly elevated in the patient myoblasts. (B) Quantification of the Western blot analysis of cytoplasmic LC3 I/II levels reveals that cytoplasmic LC3 I level was increased in the patient myoblasts. (C) Quantification of the percentage of the myoblasts with TDP-43 cytoplasmic expansion. (D) Patient-derived myoblasts were treated with 75 nM CB-5083 for 5 days, followed by nuclear and cytoplasmic fraction analysis of TDP-43 and Ubiquitin. Ubiquitin level was not changed. (E) Quantification of western blot shown in (D) revealed that TDP-43 cytoplasmic level was not changed. The experiments were performed in two patient-derived and control myoblasts. The Western blot was repeated at least twice. Statistical analysis was performed by one-way ANOVA followed by Fisher’s LSD test. [file 12967_2021_3186_MOESM1_ESM.pdf]
